# Incidence of SARS-CoV-2 reinfection among blood donors from two Brazilian states in the post-vaccination period: a prospective cohort study

**DOI:** 10.1590/S1678-9946202567033

**Published:** 2025-05-26

**Authors:** Monike Aparecida Matos de Oliveira, Tiane Sena de Castro, Renata Buccheri, Tassila Salomon, Carla Luana Dinardo, Isabel Cristina Gomes Moura, Myuki Alfaia Esashika Crispim, Nelson Abrahim Fraiji, Ester Cerdeira Sabino, Cecília Salete Alencar

**Affiliations:** 1 Universidade de São Paulo, Faculdade de Medicina, São Paulo, São Paulo, Brazil; 2 Universidade do Estado do Amazonas, Manaus, Amazonas, Brazil; 3 Vitalant Research Institute, San Francisco, California, United States; 4 Faculdade Ciências Médicas de Minas Gerais, Belo Horizonte, Minas Gerais, Brazil; 5 Fundação Pró-Sangue Hemocentro, São Paulo, São Paulo, Brazil; 6 Universidade Federal de Minas Gerais, Belo Horizonte, Minas Gerais, Brazil; 7 Fundação Hospitalar de Hematologia e Hemoterapia, Manaus, Amazonas, Brazil; 8 Universidade Municipal de São Caetano do Sul, São Caetano do Sul, São Paulo, Brazil; 9 Universidade de São Paulo, Faculdade de Medicina, Laboratório de Medicina Laboratorial (LIM-03), São Paulo, São Paulo, Brazil

**Keywords:** SARS-CoV-2, COVID-19, Reinfection, Vaccination, Immunity

## Abstract

To assess SARS-CoV-2 reinfection incidence in the post-vaccination period, we carried out a prospective cohort study of blood donors from Amazonas and Sao Paulo States, Brazil. Anti-nucleocapsid immunoglobulin (IgG anti-N) tests carried out by blood centers in 2020 were used to identify previous SARS-CoV-2 infections in blood donors and divide them into two groups: prior infection (n=386) and no prior infection (n=111). From March 2021 to January 2022, donors were followed up for six months, during which IgG anti-N and real-time reverse transcription polymerase chain reaction tests were performed every two months to detect SARS-CoV-2 infections. Symptoms and vaccination status were also recorded. Most participants (93.6%) received at least one COVID-19 vaccine dose. Reinfection incidence in the prior infection group equaled 1.39 per 100 person-months (95% CI: 0.90–2.06), in comparison to 2.68 per 100 person-months (95% CI: 1.28–4.93) for new infections in those without prior infection. The incidence risk ratio showed no significant association (0.52, 95% CI: 0.25–1.13). However, prior infection significantly increased the probability of remaining uninfected (Log-rank: p=0.009). Most reinfections (84%) showed no symptoms and occurred post-vaccination during the Delta and Omicron waves. IgG anti-N seroprevalence decreased in the prior infection group (from 35.5% at baseline to 22.5% after six months, p=0.003). Despite no significant incidence risk ratio differences, donors with prior infection had lower infection rates and a higher likelihood of remaining uninfected. Persistent post-vaccination asymptomatic infections emphasize the need for ongoing prevention, genomic surveillance, and booster programs to address emerging variants and protect vulnerable populations.

## INTRODUCTION

Brazil constituted one of the epicenters of the COVID-19 pandemic as the severe acute respiratory syndrome coronavirus 2 (SARS-CoV-2) caused over 37 million infections and 700,000 deaths^1^. From December 2020 to January 2021, nearly a year after the first recorded case in the country, relaxed social distancing measures coincided with the emergence of the Gamma variant, a new concerning variant that caused 87% of infections in January 2021, posing a significant risk of reinfection^2,3^. Consequently, the healthcare system in Manaus collapsed under a surge in new cases, a shortage of regular and Intensive Care Unit beds, and patient deaths due to oxygen shortages in hospitals^4^.

This critical period of the pandemic coincided with the beginning of the COVID-19 vaccination campaign in Brazil in January 2021^1^. Although vaccination failed to completely prevent viral infection, it played a crucial role in reducing the number of deaths, hospitalizations, and severe cases of COVID-19 worldwide, significantly contributing to pandemic control^5^. However, even in a post-vaccination context, SARS-CoV-2 reinfections still occurred^6–8^, particularly with the emergence of new variants capable of evading vaccine-induced immunity^9^. The Omicron variant has been particularly associated with a rising number of reinfections and an increased risk of third infections^10^. Furthermore, a study indicated that the protection against SARS-CoV-2 reinfection offered by vaccination and previous infection was limited^11^. These findings question the efficacy of protection against new infections in previously infected and vaccinated individuals.

Studies indicate low reinfection rates (around 1% among populations) with a low risk of severity. However, the risk of reinfection increases over longer periods after the first infection as humoral immunity declines^12^. In Brazil, data on reinfection rates in large cohorts remain limited. However, a recent study analyzing over 300,000 real-time polymerase chain reaction (RT-qPCR)-confirmed cases in multiple Brazilian states estimated a reinfection rate of approximately 8%, with a significant increase in cases following the emergence of the Omicron variant^13^.

Evidence suggests that prior infection provides some level of natural immunity that wanes over time^14,15^. Therefore, studies evaluating hybrid immunity (natural immunity from prior infection and vaccine-induced immunity) are essential to better understand the impact of infection and vaccination in the current scenario, especially with the emergence of concerning variants. Nevertheless, many cases of SARS-CoV-2 infection show milder or asymptomatic clinical manifestations, making it challenging to monitor their occurrence^16^. Thus, the use of blood donors as a study population proves effective as blood donors configure a well-known healthy group. Indeed, one of the recommendations by the World Health Organization includes blood donors as an appropriate study population for seroepidemiological investigations involving COVID-19^17^.

This study aims to analyze the incidence of SARS-CoV-2 reinfection in blood donors who were previously infected with the virus during the post-vaccination period to estimate the risk of reinfection and describe the characteristics of past cases.

## MATERIALS AND METHODS

### Study design

This prospective cohort study involved repeat blood donors from two blood centers: Fundacao Hospitalar de Hematologia e Hemoterapia do Amazonas (HEMOAM) in Manaus, Amazonas State, and Fundacao Pro-Sangue Hemocentro de Sao Paulo (FPS) in Sao Paulo State. Serological tests for SARS-CoV-2 Immunoglobulin G (IgG) anti-nucleocapsid (anti-N) antibodies were performed on samples collected during donations in 2020. Donors were categorized into two groups based on their SARS-CoV-2 infection history: prior infection and no prior infection. The prior infection group included donors with at least two donations in 2020 showing reactive IgG anti-N serology in at least one donation. The no prior infection group comprised donors with at least two donations in 2020, all of which were IgG anti-N negative and reported no previous COVID-19 diagnosis during the baseline visit. Donors with active SARS-CoV-2 infection detected by RT-qPCR at the baseline visit were excluded.

This study was carried out in Manaus from March to November 2021 and in Sao Paulo from July 2021 to January 2022. Participants were followed up for six months, with visits to the blood centers every two months. Donors in the prior infection group underwent three follow-up visits, and donors in the group with no prior infection had only two follow-up visits. This difference occurred because the recruitment of the group with no prior infection began a month after the other group.

During these visits, participants provided blood and saliva samples and completed questionnaires on clinical information such as past or current COVID-19 diagnosis, symptoms, and vaccination status. In addition to in-person visits, participants were contacted via a messaging app 30 days after each visit to fill out an electronic questionnaire. This follow-up assessed new symptoms or disease diagnoses between visits. Participants also provided sociodemographic data. The protocol visits for blood donors are shown in Supplementary Figure S1.

In Brazil, the vaccination campaign began on January 17, 2021^1^. The Brazilian National Health Surveillance Agency approved the vaccines administered to donors at the time of data collection, including CoronaVac, Oxford-AstraZeneca, Pfizer-BioNTech, and Janssen. Donors received different vaccines, and their vaccination status was verified and documented during in-person visits. The distribution of vaccines among donors is shown in Table 1.

**Table 1:** Sociodemographic characteristics and vaccination status of the study population.

Characteristics		Study group		Total cohort (n=497)	p-value
		Prior infection (n=386)	No prior infection (n=111)		
**Blood Center**					0.0188^a^
	FPS, Sao Paulo	108 (28.0%)	44 (39.6%)	152 (30.6%)	
	Hemoam, Manaus	278 (72.0%)	67 (60.4%)	345 (69.4%)	
**Sex**					0.2814^a^
	Female	105 (27.2%)	36 (32.4%)	141 (28.4%)	
	Male	281 (72.8%)	75 (67.6%)	356 (71.6%)	
**Age group (years)**					0.0251^b^
	18-29	102 (26.4%)	16 (14.4%)	118 (23.7%)	
	30-39	92 (23.8%)	28 (25.2%)	120 (24.1%)	
	40-49	115 (29.8%)	33 (29.7%)	148 (29.8%)	
	50-59	62 (16.1%)	30 (27.0%)	92 (18.5%)	
	60-69	15 (3.9%)	4 (3.6%)	19 (3.8%)	
**Self-reported skin color (n=496)**					0.0212^b^
	Asian	7 (1.8%)	6 (5.4%)	13 (2.6%)	
	White	93 (24.2%)	37 (33.3%)	130 (26.2%)	
	Black	46 (11.9%)	14 (12.6%)	60 (12.1%)	
	Mixed ethnicity	239 (62.1%)	54 (48.6%)	293 (59.1%)	
**Level of education (n=495)**					0.9633^b^
	Primary Education	16 (4.2%)	5 (4.5%)	21 (4.2%)	
	Secondary Education	142 (37.0%)	40 (36.0%)	182 (36.8%)	
	Tertiary Education	226 (58.9%)	66 (59.5%)	292 (59.0%)	
**Monthly income^c^ (n=484)**					0.0410^b^
	None	31 (8.2%)	11 (10.2%)	42 (8.7%)	
	1–2 minimum wages	186 (49.5%)	42 (38.9%)	228 (47.1%)	
	3–4 minimum wages	86 (22.9%)	21 (19.4%)	107 (22.1%)	
	5–10 minimum wages	61 (16.2%)	25 (23.1%)	86 (17.8%)	
	>10 minimum wages	12 (3.2%)	9 (8.3%)	21 (4.3%)	
**Vaccination status (n=471)**					0.0270^b^
	Yes (at least one dose)	334 (92.3%)	107 (98.2%)	441 (93.6%)	
	No	28 (7.7%)	2 (1.8%)	30 (6.4%)	
**Vaccine (n=441)**					0.2899^b^
	Oxford-AstraZeneca	150 (44.9%)	57 (53.3%)	207 (46.9%)	
	CoronaVac	115 (34.4%)	36 (33.6%)	151 (34.2%)	
	Janssen	10 (3.0%)	1 (0.9%)	11 (2.5%)	
	Pfizer-BioNTech	59 (17.7%)	13 (12.1%)	72 (16.3%)	
**Doses (n=441)**					0.0001^b^
	One dose	50 (15.0%)	2 (1.9%)	52 (11.8%)	
	Two doses	274 (82.0%)	104 (97.2%)	378 (85.7%)	
	Single dose	10 (3.0%)	1 (0.9%)	11 (2.5%)	
**Additional dose (n=441)**					0.0004^b^
	Oxford-AstraZeneca	3 (0.9%)	1 (0.9%)	4 (0.9%)	
	Janssen	2 (0.6%)	0 (0%)	2 (0.5%)	
	Pfizer-BioNTech	47 (14.1%)	34 (31.8%)	81 (18.4%)	
None		282 (84.4%)	72 (67.3%)	354 (80.3%)	
**Donors under follow-up during circulation of predominant variants^d^**					< 0.0001^b^
	Gamma (Apr-Jul 2021)	298 (42.8%)	0 (0%)	298 (36.3%)	
	Delta (Aug-Dec 2021)	349 (50.1%)	111 (88.1%)	460 (56%)	
	Omicron (January 2022)	49 (7%)	15 (11.9%)	64 (7.8%)	

a Pearson's chi-squared test;

b Fisher's exact test;

c Brazilian minimum wage in 2021;

d Counts represent participants who were actively followed during the predominant circulation period of each variant. Participants may be under follow-up during more than one period.

The Brazilian Research Ethics Committee and the Institutional Review Boards of the Hospital das Clinicas da Faculdade de Medicina da Universidade de Sao Paulo and participating blood banks reviewed and approved this study (CAAE N° 39790920.9.1001.0009). The study was explained to all participants, who signed informed consent agreeing to participate.

### Sample testing for SARS-CoV-2

The collected blood samples were tested by an automated chemiluminescent microparticle immunoassay (SARS-CoV-2 IgG, Abbott Laboratories, Abbott Park, IL, USA; reference 6R8620) to detect IgG antibodies against the SARS-CoV-2 nucleocapsid (N) protein in serum samples, with a cutoff value for positive samples of 1.4 Index (S/C). The specificity reported by the manufacturer equaled 99.63% and the sensitivity, 86.36% after seven days from the onset of COVID-19 symptoms (100% after 14 days).

Saliva samples were used for the molecular detection of the virus. Nucleic acid extraction was performed using the Extracta Kit Fast – DNA and RNA Viral (MVXA-P096 FAST) on an automated EXTRACTA 96 extractor (Loccus do Brasil LTDA, Cotia, SP, BR). The success of the extraction was confirmed by quantifying the genetic material. Subsequently, the Allplex^™^ SARS-CoV-2/FluA/FluB/RSV multiplex assay, a real-time reverse transcription polymerase chain reaction, was conducted to detect and differentiate the N, RdRP, and S genes of SARS-CoV-2 (Seegene Inc, Seoul, KR). The protocol was followed according to the instructions for the extraction-free method for saliva samples as the assay manufacturer reported the 100% analytical sensitivity of this method.

### Criteria for identifying SARS-CoV-2 infections

Serological and molecular tests were conducted on donor samples during all in-person visits to identify incident SARS-CoV-2 infections during follow-up. Incident infections were confirmed under the following conditions:

1. Positive RT-qPCR Test: Participants who tested positive for SARS-CoV-2 via RT-qPCR in any study visit sample.
2. Seroconversion of IgG anti-N Antibodies: Participants with negative RT-qPCR results but evidence of seroconversion of IgG anti-N antibodies between visits. Seroconversion was defined as a negative IgG anti-N result at one visit followed by a positive result at the subsequent visit. The incident infection date was estimated as the midpoint between the two visits.
3. Significant Increase in Antibody Titers: Participants in the prior infection group who had a positive IgG anti-N result at the baseline visit and showed a significant increase in antibody titers (>1.0 Index [S/C]) between visits despite negative RT-qPCR results. The date of incident infection was similarly estimated as the midpoint between the two visits.

Serological tests for participants who reported receiving the CoronaVac vaccine were analyzed considering vaccination dates. If seroconversion or an increase in antibody titers occurred within three months of any CoronaVac dose, the event was classified as a vaccine response rather than an incident infection. This distinction accounts for the inactivated virus platform of CoronaVac, which induces the production of IgG anti-N antibodies.

### Classification of serological results and infections

Donors in the prior infection group who experienced an incident infection during the study, confirmed by a positive RT-qPCR or seroconversion/increased IgG anti-N titers, were classified as reinfections. Participants in the group without prior infection who had an incident infection confirmed by the same criteria were classified as new infections.

### Statistical analysis

Qualitative variables are shown as frequencies and percentages. The association between qualitative variables was assessed using Pearson's chi-squared and Fisher's exact tests. Quantitative variables are shown as median and interquartile range (IQR), and their association was analyzed using the two-tailed Mann-Whitney test.

The incidence rates of SARS-CoV-2 infection are shown per 100 person-months, considering the follow-up time of each participant. In total, 95% confidence intervals were calculated using the exact Poisson distribution method for proportions. Survival analysis was performed by the Kaplan-Meier method, with survival probability shown with their 95% confidence intervals. Differences between survival curves were assessed by the log-rank test (Mantel-Haenszel test). Analyses were conducted on R, version 4.2.0, and a p<0.05 was considered statistically significant.

## RESULTS

This study recruited 501 blood donors, excluding four of them due to active SARS-CoV-2 infection confirmed by positive saliva RT-qPCR at the baseline visit. The final cohort consisted of 497 participants, with 386 (77.7%) in the prior infection group and 111 (22.3%) in the no prior infection group. Table 1 describes the sociodemographic characteristics and vaccination status of the cohort. Most donors received the Oxford-AstraZeneca (46.9%) or CoronaVac (34.2%) vaccines, with a significant portion of unvaccinated individuals belonging to the prior infection group.

A total of 35 donors experienced incident SARS-CoV-2 infections during follow-up. Of these, 25 cases occurred in the prior infection group, corresponding to a reinfection incidence of 1.39 per 100 person-months (95% CI: 0.90–2.06). The no prior infection group included 10 cases of new infection, yielding a higher incidence of 2.68 per 100 person-months (95% CI: 1.28–4.93). The results stratified by blood center also showed the same trend, with the incidence estimate for the no prior infection group exceeding that for the prior infection group in both centers. The incidence risk ratio (IRR) of new SARS-CoV-2 infection among donors with prior infection, when compared to those without it, equaled 0.52 (95% CI: 0.25–1.13), indicating no statistically significant association (Table 2). Notably, participants showed a positive RT-qPCR result or seroconversion/increased antibody titers only once during this study.

**Table 2:** Incidence density of SARS-CoV-2 infection in the cohort.

Blood Center	Study group	N° of donors	N° of infections	Follow-up time (person-months)	Incidence per 100 person-months (95% CI)	Incidence risk ratio (95% CI)
FPS, Sao Paulo	Prior Infection	108	12	431.87	2.78 (1.44–4.85)	0.58 (0.23–1.59)
	No prior infection	44	7	147.87	4.73 (1.90–9.75)	
Hemoam, Manaus	Prior Infection	278	13	1,360.17	0.96 (0.51–1.63)	0.69 (0.22–3.15)
	No prior infection	67	3	225.37	1.33 (0.27–3.89)	
Total cohort	Prior Infection	386	25	1,792.03	1.39 (0.90–2.06)	0.52 (0.25–1.13)
	No prior infection	111	10	373.23	2.68 (1.28–4.93)	
	Overall	497	35	2,165.26	1.62 (1.13–2.25)	–

**Figure 1:**
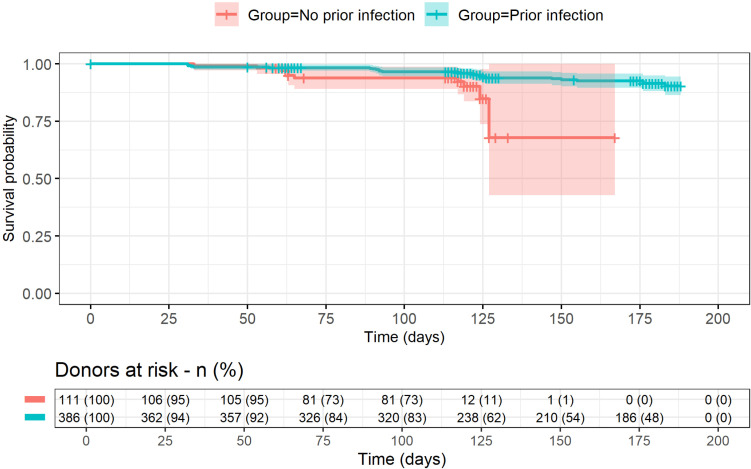
Kaplan-Meier survival curves by study group. Survival probability is shown with 95% confidence intervals as colored boxes alongside the lines. Vertical marks represent censored observations (Lost to Follow-up).

The Kaplan-Meier survival analysis indicated a significant difference between the prior infection and no prior infection groups regarding the time up to the SARS-CoV-2 incident infections during follow-up. The prior infection group showed a higher probability of no SARS-CoV-2 infection during follow-up (p = 0.009, Chi-square = 6.8) (Figure 1).

The epidemiological profile of blood donors with incident SARS-CoV-2 infections was similar across study groups, except for the method of infection detection. In the reinfection group (prior infection group), RT-qPCR detected 44% of cases, compared to 90% in the new infection group (no prior infection group), a significant difference (p=0.022). Donors with reinfections were predominantly male (52.0%) aged under 50 years who received CoronaVac doses (48.0%). Most infections (60.0%) occurred after the primary vaccination series, although two unvaccinated individuals also underwent reinfections. Reinfection cases were largely asymptomatic (84%), unlike first infections, in which 60% of patients reported symptoms. However, confirmatory testing to determine if the symptoms were due to SARS-CoV-2 infection only occurred in six cases (Table 3).

**Table 3:** Epidemiological profile of blood donors with incident SARS-CoV-2 infection in the cohort.

Characteristics		Study group		Total infected (n=35)	p-value^a^
		Prior infection (n=25)	No prior infection (n=10)		
**Blood Center**					0.2853
	FPS, Sao Paulo	12 (48.0%)	7 (70.0%)	19 (54.3%)	
	Hemoam, Manaus	13 (52.0%)	3 (30.0%)	16 (45.7%)	
**Sex**					0.7233
	Female	12 (48.0%)	4 (40.0%)	16 (45.7%)	
	Male	13 (52.0%)	6 (60.0%)	19 (54.3%)	
**Age group (years)**					0.1999
	18-29	7 (28.0%)	0 (0%)	7 (20.0%)	
	30-39	6 (24.0%)	3 (30.0%)	9 (25.7%)	
	40-49	7 (28.0%)	2 (20.0%)	9 (25.7%)	
	50-59	3 (12.0%)	4 (40.0%)	7 (20.0%)	
	60-69	2 (8.0%)	1 (10.0%)	3 (8.6%)	
**Self-reported skin color**					0.1604
	Asian	0 (0%)	0 (0%)	0 (0%)	
	White	11 (44.0%)	8 (80.0%)	19 (54.3%)	
	Black	1 (4.0%)	0 (0%)	1 (2.9%)	
	Mixed ethnicity	13 (52.0%)	2 (20.0%)	15 (42.9%)	
**Level of education**					0.7452
	Primary Education	3 (12.0%)	0 (0%)	3 (8.6%)	
	Secondary Education	10 (40.0%)	5 (50.0%)	15 (42.9%)	
	Higher Education	12 (48.0%)	5 (50.0%)	17 (48.6%)	
**Monthly income^b^ (n=34)**					0.3132
	None	2 (8.0%)	1 (11.1%)	3 (8.8%)	
	1–2 minimum wages	15 (60.0%)	3 (33.3%)	18 (52.9%)	
	3–4 minimum wages	3 (12.0%)	2 (22.2%)	5 (14.7%)	
	5–10 minimum wages	5 (20.0%)	2 (22.2%)	7 (20.6%)	
	>10 minimum wages	0 (0%)	1 (11.1%)	1 (2.9%)	
**Vaccine**					0.9186
	Oxford-AstraZeneca	4 (16.0%)	2 (20.0%)	6 (17.1%)	
	CoronaVac	12 (48.0%)	4 (40.0%)	16 (45.7%)	
	Janssen	1 (4.0%)	1 (10.0%)	2 (5.7%)	
	Pfizer-BioNTech	6 (24.0%)	3 (30.0%)	9 (25.7%)	
	Unvaccinated	2 (8.0%)	0 (0%)	2 (5.7%)	
**Method of infection detectio**n					0.022
	RT-qPCR	11 (44.0%)	9 (90.0%)	20 (57.1%)	
	Serological test	14 (56.0%)	1 (10.0%)	15 (42.9%)	
**Moment of infection**					0.3556
	Before the first dose	4 (16.0%)	0 (0%)	4 (11.4%)	
	After second dose	15 (60.0%)	8 (80.0%)	23 (65.7%)	
	After single dose	0 (0%)	1 (10.0%)	1 (2.9%)	
	After additional dose	4 (16.0%)	1 (10.0%)	5 (14.3%)	
	Unvaccinated	2 (8.0%)	0 (0%)	2 (5.7%)	
**Number of infections during circulation of predominant variants**					0.0887
	Gamma (Apr-Jul 2021)	9 (36.0%)	0 (0%)	9 (25.7%)	
	Delta (Aug-Dec 2021)	10 (40.0%)	7 (70.0%)	17 (48.6%)	
	Omicron (January 2022)	6 (24.0%)	3 (30.0%)	9 (25.7%)	
**Symptoms in the current infection**					1
	Yes	4 (16.0%)	1 (10.0%)	5 (14.3%)	
	No	21 (84.0%)	9 (90.0%)	30 (85.7%)	
**Symptoms in previous infection (n=25)**					-
	Yes, with a confirmatory test	6 (24.0%)	-	-	
	Yes, without a confirmatory test	9 (36.0%)	-	-	
	No	10 (40.0%)	-	-	

a Fisher's exact test;

b Brazilian minimum wage in 2021.

The most reported symptoms in symptomatic donors with incident infection (n=5) referred to headaches (5/5), fatigue (4/5), and body aches (4/5). Other manifestations included fever (2/5), chills (2/5), cough (3/5), shortness of breath (1/5), loss of taste (1/5), loss of smell (1/5), sore throat (3/5), rhinorrhea (3/5), and nausea (1/5). The infections observed in the cohort mostly occurred in October 2021 (n=8) and January 2022 (n=9) (Supplementary Figure S2).

**Figure 2:**
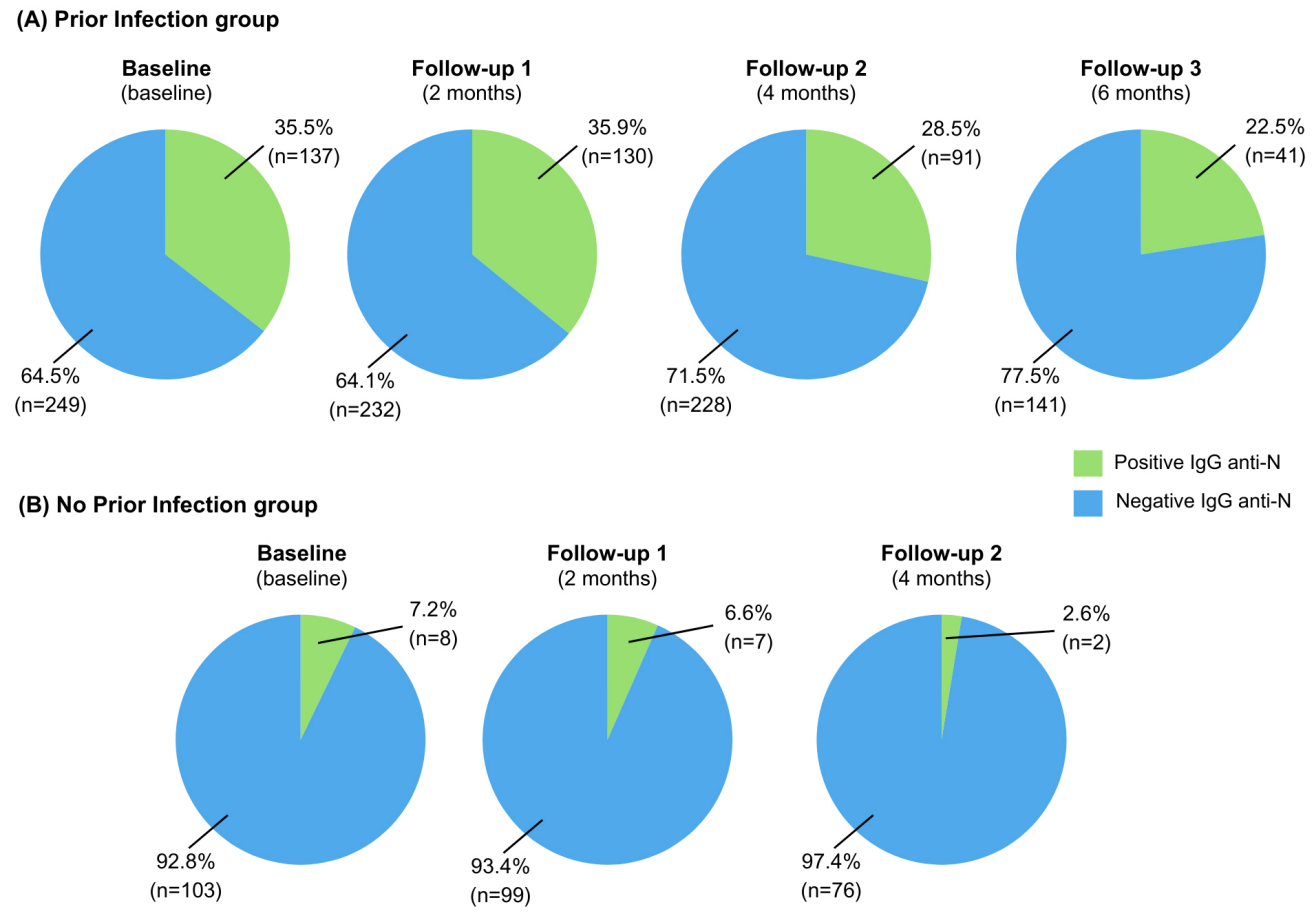
Seroprevalence of SARS-CoV-2 IgG anti-N antibodies in blood donors during study visits: (A) Prior infection group; (B) No prior infection group. Green and blue represent positive and negative results in the SARS-CoV-2 IgG anti-N chemiluminescent microparticle immunoassay, respectively. Values are shown in proportions of donors with samples collected at each visit. No samples were collected at follow-up three for the group without prior infection.

Regarding anti-N IgG antibodies, at baseline, 35.5% of donors in the prior infection group had positive antibodies. By the end of follow-up, only 22.5% of donors with prior infection still had detectable antibodies, indicating a decrease in seroprevalence over time for this group (p=0.003) (Figure 2A). In the group without prior infection, all participants had negative baseline anti-N antibodies, except for 7.2% of donors who had been vaccinated with CoronaVac. Throughout the follow-up period, seroprevalence was also related to CoronaVac vaccination. Only one donor in this group tested positive for anti-N due to an incident SARS-CoV-2 infection at follow-up 1. Seroprevalence over time for the no prior infection group showed no significant variations (p=0.3617) (Figure 2B).

Assessing only donors with incident SARS-CoV-2 infection during this study (n=35) showed that 76% (n=19) of infected participants in the prior infection group tested positive for IgG anti-N antibodies following infection, of whom 26.3% (n=5) had pre-existing positive antibodies. In contrast, in the no prior infection group, only one donor (10%) tested positive for IgG anti-N antibodies post-infection, having received the CoronaVac vaccine. No donor in the no prior infection group had pre-existing positive antibodies before infection. Also, hybrid immunity influenced anti-N antibody titers in vaccinated individuals with incident infections in the cohort (n=33). Levels in the prior infection group significantly exceeded those in the no prior infection group in the whole sample and for CoronaVac and other vaccines before and after infection (p<0.05). Donors with prior infection who received the CoronaVac vaccine showed higher titers than those in the same group who received other vaccines. Additionally, donors without prior infection showed low levels of anti-N antibodies before and after infection regardless of whether they had received CoronaVac or other vaccines (Table 4).

**Table 4:** Anti-N IgG titers in vaccinated blood donors with incident SARS-CoV-2 infection.

		Study group		p-value
		Prior infection	No prior infection	
**Total sample**	**Donors (n)**	23	10	
	**Median anti-N IgG – IQR^a^**			
	Before infection	0.63–1.64	0.07–0.07	< 0.0001
	After infection	1.95–3.97	0.08–0.16	< 0.0001
**CoronaVac**	**Donors (n)**	12	4	
	**Median anti-N IgG – IQR**			
	Before infection	1.48–1.35	0.05–0.18	0.001829
	After infection	2.19–2.54	0.29–0.17	0.00609
**Other vaccines**	**Donors (n)**	11	6	
	**Median anti-N IgG – IQR**			
	Before infection	0.18–0.49	0.08–0.07	0.01145
	After infection	1.82 – 4.75	0.07–0.02	0.000668

a Median and interquartile range (IQR) values calculated using the results of the SARS-CoV-2 IgG anti-N chemiluminescent microparticle immunoassay and stratified according to the date of infection observed during study follow-up.

## DISCUSSION

This cohort study investigated SARS-CoV-2 reinfection rates among blood donors by comparing individuals with prior SARS-CoV-2 infections to those with no prior infections. Our study found that the rates for reinfections in donors with prior infection were lower than the rates for new infections in previously uninfected donors. Nevertheless, infection IRR failed to statistically differ between the two groups (IRR = 0.52, 95% CI: 0.25 - 1.13). Interestingly, Kaplan-Meier survival analysis showed that individuals with prior infections had a significantly higher probability of remaining uninfected during the follow-up in this study.

Observational studies in England^18^ and Denmark^19^ and a meta-analysis of 11 cohort studies^20^ reported findings similar to those in our study, showing lower rates of SARS-CoV-2 reinfection than of new infections. However, these studies differed from ours by associating a significantly lower risk of infection among individuals with prior exposure. That meta-analysis, on studies from January 2020 to April 2021, found a substantially reduced risk of reinfection (hazard ratio=0.12, 95% CI 0.09–0.17) for previously infected individuals^20^. Several factors may explain the discrepancy between our IRR results and those of other studies. Our research had a smaller sample size and challenges in long-term participant follow-up, which could have affected our results. Additionally, our cohort had a high vaccination rate (93.6%), unlike other studies. The timing of the studies is also crucial as the referenced studies had been carried out before the emergence of Delta and Omicron variants, potentially affecting the risk of reinfection. Furthermore, variations in vaccination rates and predominant circulating variants in different study periods may influence the risk of reinfection among previously infected individuals. These factors highlight the complexity of comparing reinfection risks across studies and underscore the need for ongoing research to account for evolving viral variants and vaccination status.

Our study provides valuable insights into SARS-CoV-2 infection dynamics within a healthy population during the circulation of the concerning Delta and Omicron variants, coinciding with the progress of COVID-19 vaccination. Although we were unable to sequence the samples with incident infections, data from the Fiocruz Genomics Network enabled us to infer the likely involved variants. The donors selected for the prior infection group had been infected in 2020, when the main circulating variants in Brazil were B1.1.28, B1.1.33, B1.1, and P2. On the other hand, most incident infections in our cohort occurred during two periods: October 2021 (characterized by increased circulation of the Delta variant) and January 2022 (marked by the predominance of the Omicron variant)^21^. Importantly, these incident infections coincided with a period in which a substantial portion of the Brazilian adult population had received at least one dose of COVID-19 vaccines. This context shows the interplay between emerging variants and vaccine-induced immunity in a real-world setting. Our study has a particularly relevant timing as it captures the transition from Delta to Omicron dominance in Brazil.

The combination of high vaccination rates and the emergence of these highly transmissible variants provides an opportunity to assess the impact of hybrid immunity (from prior infections and vaccination) on SARS-CoV-2 reinfection rates. Studies on reinfection rates during the Delta and Omicron waves have shown an increase in relation to pre-variant periods^22^. Notably, most reinfections occurred during the Omicron transmission period^23,24^, a trend also present in our research. The increased reinfection rates during the Omicron wave, despite widespread vaccination and prior infections, underscore the capacity of the virus to adapt and challenge acquired immunity.

The epidemiological profile of donors with incident infections in our study showed consistent patterns across groups. Reinfections predominantly occurred in men and adults aged under 50 years, as in findings from other observational studies^12,25^. Notably, most reinfections in our cohort occurred in fully vaccinated individuals, with a significant proportion of them having received CoronaVac. This trend mirrors an epidemiological survey that reported most reinfections in individuals vaccinated with Sinopharm, which uses the same inactivated virus technology as CoronaVac^12^. These observations highlight a crucial point: while prior infection and vaccination offer a degree of protection, they fail to confer complete immunity against new infections. The risk of reinfection persists, potentially exacerbated by immune evasion capabilities of emerging variants. Evidence showing that the neutralizing response induced by vaccines against principal virus variants wanes over time supports this finding^26^.

Despite the ongoing risk of reinfection, our study showed a low severity profile for incident infections. In our cohort, most reinfections were asymptomatic or mild, in line with findings from other studies examining reinfection cases^27–29^. This reduced severity can be attributed to hybrid immunity, which seems more robust and durable than immunity from vaccination or infection alone. Hybrid immunity combines protection against the S antigen stimulated by vaccination (in mRNA and adenoviral vector vaccines) with humoral and cellular responses against other viral antigens, such as M and N antigens, conferred by natural immunity following infection^30^.

Specifically, T cell-mediated immune responses seem to play a crucial role in protecting against severe disease. Despite the decrease in neutralizing antibody response against the Omicron variant, studies indicate that vaccination induces a highly conserved cellular immunity against severe manifestations of Omicron infection^31^. Keeton *et al*.^32^ showed that cellular response remained conserved across major variants, with about from 70 to 80% of CD4+ and CD8+ T cell responses persisted in vaccinated and previously infected individuals, showing a similar magnitude of cross-reactivity against Beta (B.1.351), Delta (B.1.617.2) and Omicron variants. Furthermore, individuals with prior infection who were vaccinated produce more specific memory B cells, targeting the RBD region and neutralizing antibodies against variants, along with a unique profile of CD4+ T cell cytokines^33^.

Our assessment of IgG anti-N antibodies found a decline in seroprevalence among the prior infection group, from 35.5% at baseline to 22.5% by its end. This trend aligns with findings from studies in Japan, Finland, Thailand, and England, which reported decreasing anti-N antibody positivity over time, particularly in young adults and asymptomatic individuals^34–37^. When examining vaccinated donors with incident infections, we found that those with prior infection (hybrid immunity) showed higher levels of IgG anti-N antibodies than those without prior infection. Interestingly, CoronaVac vaccination seemed to stimulate higher anti-N antibody production only in individuals with prior infection. This difference in anti-N antibody production may be explained by the tendency of CoronaVac to stimulate higher anti-S than anti-N antibody production in individuals without prior infection. A study on healthcare providers who had been vaccinated with CoronaVac without a history of prior infections reported 96.2% positivity for anti-S antibodies but only 51.2% for anti-N antibodies. On the other hand, 98.8% of unvaccinated individuals with SARS-CoV-2 infection tested positive for anti-N antibodies shortly after infection. These findings suggest that higher anti-N antibody production may be associated with prior infection followed by vaccination rather than vaccination alone^38^. Additionally, anti-N antibody titers can vary according to disease severity. Mild cases typically show lower anti-N titers than severe cases, which produce a more robust antibody response associated with higher viral loads during infection^39,40^. Therefore, the moderate anti-N titers in our cohort may be attributed to the predominance of mild cases.

The main strengths of our study include its frequent serological and molecular testing of participants, which can detect asymptomatic infections, and follow-up questionnaires. However, limitations such as its small sample size and participant attrition may affect its estimate accuracy despite relevant trends. The study population, consisting of blood donors with higher education levels, may have better access to and knowledge of preventive measures, potentially influencing exposure risk behavior and behavioral changes in the prior infection group. A notable challenge referred to detecting incident infections by IgG anti-N testing in CoronaVac-vaccinated participants as it may have missed infections occurring close to vaccination. This study mitigated this risk by RT-qPCR testing alongside serological examination. The concurrent vaccination of participants in this study hindered better stratification of donors by vaccine type. Future research should individually assess the effect of each vaccine on reinfection rates.

## CONCLUSIONS

The current widespread SARS-CoV-2 exposure by infection or vaccination tends toward disease stabilization with predominantly mild manifestations. However, our data indicate that infections continue to occur, particularly in healthy populations such as the blood donors in our cohort. These individuals often experience asymptomatic infections, potentially becoming silent carriers of the virus. Therefore, it remains essential to continue promoting prevention measures, maintain viral genomic surveillance, and implement periodic vaccination programs, including booster doses targeting emerging concerning variants regardless of prior infection history. These ongoing efforts are crucial to manage the evolving nature of SARS-CoV-2 and protect vulnerable populations.

## Supplementary Material



## References

[1] World Health Organization WHO COVID-19 dashboard.

[2] Faria NR, Mellan TA, Whittaker C, Claro IM, Candido DD, Mishra S (2021). Genomics and epidemiology of the P.1 SARS-CoV-2 lineage in Manaus, Brazil. Science.

[3] Prete CA, Buss LF, Buccheri R, Abrahim CM, Salomon T, Crispim MA (2022). Reinfection by the SARS-CoV-2 Gamma variant in blood donors in Manaus, Brazil. BMC Infect Dis.

[4] Barreto IC, Costa RV, Ramos RF, Oliveira LG, Martins NR, Cavalcante FV (2021). Health collapse in Manaus: the burden of not adhering to non-pharmacological measures to reduce the transmission of Covid-19. Saude Debate.

[5] Fiolet T, Kherabi Y, MacDonald CJ, Ghosn J, Peiffer-Smadja N (2022). Comparing COVID-19 vaccines for their characteristics, efficacy and effectiveness against SARS-CoV-2 and variants of concern: a narrative review. Clin Microbiol Infect.

[6] Hall V, Foulkes S, Insalata F, Kirwan P, Saei A, Atti A (2022). Protection against SARS-CoV-2 after Covid-19 vaccination and previous infection. N Engl J Med.

[7] Cohen JI, Burbelo PD (2021). Reinfection with SARS-CoV-2: implications for vaccines. Clin Infect Dis.

[8] Harrington D, Kele B, Pereira S, Couto-Parada X, Riddell A, Forbes S (2021). Confirmed reinfection with severe acute respiratory syndrome Coronavirus 2 (SARS-CoV-2) variant VOC-202012/01. Clin Infect Dis.

[9] Keeling MJ (2023). Patterns of reported infection and reinfection of SARS-CoV-2 in England. J Theor Biol.

[10] Pulliam JR, van Schalkwyk C, Govender N, von Gottberg A, Cohen C, Groome MJ (2022). Increased risk of SARS-CoV-2 reinfection associated with emergence of Omicron in South Africa. Science.

[11] Yu W, Guo Y, Hu T, Liu Y, Fan Q, Guo L (2023). Incidence and severity of SARS-CoV-2 reinfection, a multicenter cohort study in Shanghai, China. J Med Virol.

[12] Almadhi M, Alsayyad AS, Conroy R, Atkin S, Awadhi AA, Al-Tawfiq JA (2022). Epidemiological assessment of SARS-CoV-2 reinfection. Int J Infect Dis.

[13] Fonseca PL, Malta FS, Braga-Paz I, Silva JP, Souza CS, Aguiar RS (2024). SARS-CoV-2 reinfection rate before and after VOC Omicron emergence: a retrospective study in Brazil. Braz J Microbiol.

[14] Lewis N, Chambers LC, Chu HT, Fortnam T, De Vito R, Gargano LM (2022). Effectiveness associated with vaccination after COVID-19 recovery in preventing reinfection. JAMA Netw Open.

[15] Sabino EC, Buss LF, Carvalho MP, Prete CA, Crispim MA, Fraiji NA (2021). Resurgence of COVID-19 in Manaus, Brazil, despite high seroprevalence. Lancet.

[16] van Eijk LE, Binkhorst M, Bourgonje AR, Offringa AK, Mulder DJ, Bos EM (2021). COVID-19: immunopathology, pathophysiological mechanisms, and treatment options. J Pathol.

[17] Coyne D, Butler D, Meehan A, Keogh E, Williams P, Carterson A (2023). The changing profile of SARS-CoV-2 serology in Irish blood donors. Glob Epidemiol.

[18] Hall VJ, Foulkes S, Charlett A, Atti A, Monk EJ, Simmons R (2021). SARS-CoV-2 infection rates of antibody-positive compared with antibody-negative health-care workers in England: a large, multicentre, prospective cohort study (SIREN). Lancet.

[19] Hansen CH, Michlmayr D, Gubbels SM, Mølbak K, Ethelberg S (2021). Assessment of protection against reinfection with SARS-CoV-2 among 4 million PCR-tested individuals in Denmark in 2020: a population-level observational study. Lancet.

[20] Deng L, Li P, Zhang X, Jiang Q, Turner D, Zhou C (2022). Risk of SARS-CoV-2 reinfection: a systematic review and meta-analysis. Sci Rep.

[21] Brasil Ministério da Saúde. Fundação Oswaldo Cruz. Dashboard genomic network.

[22] Jang EJ, Choe YJ, Yun GW, Wang S, Cho UJ, Yi S (2022). Reinfection with SARS-CoV-2 in general population, South Korea; nationwide retrospective cohort study. J Med Virol.

[23] Nguyen NN, Nguyen YN, Hoang VT, Million M, Gautret P (2023). SARS-CoV-2 reinfection and severity of the disease: a systematic review and meta-analysis. Viruses.

[24] Guedes AR, Oliveira MS, Tavares BM, Luna-Muschi A, Lazari CS, Montal AC (2023). Reinfection rate in a cohort of healthcare workers over 2 years of the COVID-19 pandemic. Sci Rep.

[25] Al-Otaiby M, Krissaane I, Al Seraihi A, Alshenaifi J, Qahtani MH, Aljeri T (2022). SARS-CoV-2 reinfection rate and outcomes in Saudi Arabia: a national retrospective study. Int J Infect Dis.

[26] Evans JP, Zeng C, Carlin C, Lozanski G, Saif LJ, Oltz EM (2022). Neutralizing antibody responses elicited by SARS-CoV-2 mRNA vaccination wane over time and are boosted by breakthrough infection. Sci Transl Med.

[27] Cegolon L, Magnano G, Negro C, Larese Filon F (2023). SARS-CoV-2 reinfections in health-care workers, 1 March 2020-31 January 2023. Viruses.

[28] Morris CP, Eldesouki RE, Fall A, Gaston DC, Norton JM, Gallagher ND (2022). SARS-CoV-2 reinfections during the Delta and Omicron waves. JCI Insight.

[29] Deng J, Ma Y, Liu Q, Du M, Liu M, Liu J (2023). Severity and outcomes of SARS-CoV-2 reinfection compared with primary infection: a systematic review and meta-analysis. Int J Environ Res Public Health.

[30] Lasrado N, Barouch DH (2023). SARS-CoV-2 hybrid immunity: the best of both worlds. J Infect Dis.

[31] Liu J, Chandrashekar A, Sellers D, Barrett J, Jacob-Dolan C, Lifton M (2022). Vaccines elicit highly conserved cellular immunity to SARS-CoV-2 Omicron. Nature.

[32] Keeton R, Tincho MB, Ngomti A, Baguma R, Benede N, Suzuki A (2022). T cell responses to SARS-CoV-2 spike cross-recognize Omicron. Nature.

[33] Rodda LB, Morawski PA, Pruner KB, Fahning ML, Howard CA, Franko N (2022). Imprinted SARS-CoV-2-specific memory lymphocytes define hybrid immunity. Cell.

[34] Matsuba I, Takuma T, Hatori N, Takai M, Watanabe Y, Takada N (2022). The proportion of long-term response to anti-N IgG antibody after 12 months for COVID-19 subclinical infections and a longitudinal survey for COVID-19 subclinical infections in 2021. Intern Med.

[35] Haveri A, Ekström N, Solastie A, Virta C, Österlund P, Isosaari E (2021). Persistence of neutralizing antibodies a year after SARS-CoV-2 infection in humans. Eur J Immunol.

[36] Chansaenroj J, Yorsaeng R, Posuwan N, Puenpa J, Wanlapakorn N, Sudhinaraset N (2021). Long-term specific IgG response to SARS-CoV-2 nucleocapsid protein in recovered COVID-19 patients. Sci Rep.

[37] Krutikov M, Palmer T, Tut G, Fuller C, Azmi B, Giddings R (2022). Prevalence and duration of detectable SARS-CoV-2 nucleocapsid antibodies in staff and residents of long-term care facilities over the first year of the pandemic (VIVALDI study): prospective cohort study in England. Lancet Healthy Longev.

[38] Lumley SF, Wei J, O’Donnell D, Stoesser NE, Matthews PC, Howarth A (2021). The duration, dynamics, and determinants of severe acute respiratory syndrome Coronavirus 2 (SARS-CoV-2) antibody responses in individual healthcare workers. Clin Infect Dis.

[39] Azak E, Karadenizli A, Uzuner H, Karakaya N, Canturk NZ, Hulagu S (2021). Comparison of an inactivated Covid19 vaccine-induced antibody response with concurrent natural Covid19 infection. Int J Infect Dis.

[40] Zhang X, Lu S, Li H, Wang Y, Lu Z, Liu Z (2020). Viral and antibody kinetics of COVID-19 patients with different disease severities in acute and convalescent phases: a 6-month follow-up study. Virol Sin.

