# Supplementary material for: Incidence of SARS-CoV-2 reinfection among blood donors from two Brazilian states in the post-vaccination period: a prospective cohort study
Source: Rev Inst Med Trop Sao Paulo. 2025 May 26;67:e33. doi: 10.1590/S1678-9946202567033 (PMC12105851; doi:10.1590/S1678-9946202567033)
Supplement: Supplementary file 1 [file 1678-9946-rimtsp-67-S1678-9946202567033-suppl1.pdf]

R E V I S T A
DO
INSTITUTO
DE
MEDICINA
TROPICAL
DE
SÃO PAULO

JOURNAL OF THE SÃO PAULO INSTITUTE OF TROPICAL MEDICINE

SUPPLEMENTARY MATERIAL

https://doi.org/10.48331/scielodata.VTTDDU

Incidence of SARS-CoV-2 reinfection among blood donors from two Brazilian states in the post-vaccination period: a prospective cohort study

Monike Aparecida Matos de Oliveira [1], Tiane Sena de Castro [2], Renata Buccheri [3], Tassila Salomon [4], Carla Luana Dinardo [5], Isabel Cristina Gomes Moura [6], Myuki Alfaia Esashika Crispim [7], Nelson Abrahim Fraiji [7], Ester Cerdeira Sabino [1,8], Cecília Salete Alencar [9]

[1]Universidade de São Paulo, Faculdade de Medicina, São Paulo, São Paulo, Brazil

[2]Universidade do Estado do Amazonas, Manaus, Amazonas, Brazil

[3]Vitalant Research Institute, San Francisco, California, United States

[4]Faculdade Ciências Médicas de Minas Gerais, Belo Horizonte, Minas Gerais, Brazil

[5]Fundação Pró-Sangue Hemocentro, São Paulo, São Paulo, Brazil

[6]Universidade Federal de Minas Gerais, Belo Horizonte, Minas Gerais, Brazil

[7]Fundação Hospitalar de Hematologia e Hemoterapia, Manaus, Amazonas, Brazil

[8]Universidade Municipal de São Caetano do Sul, São Caetano do Sul, São Paulo, Brazil

[9]Universidade de São Paulo, Faculdade de Medicina, Laboratório de Medicina Laboratorial (LIM-03), São Paulo, São Paulo, Brazil

Correspondence to: Monike Aparecida Matos de Oliveira
Universidade de São Paulo, Faculdade de Medicina, Av. Dr. Arnaldo, 455, Cerqueira César. CEP 01246-903, São Paulo, SP, Brazil

E-mail: olliveira.monike@gmail.com

Received: 7 January 2025

Accepted: 21 March 2025

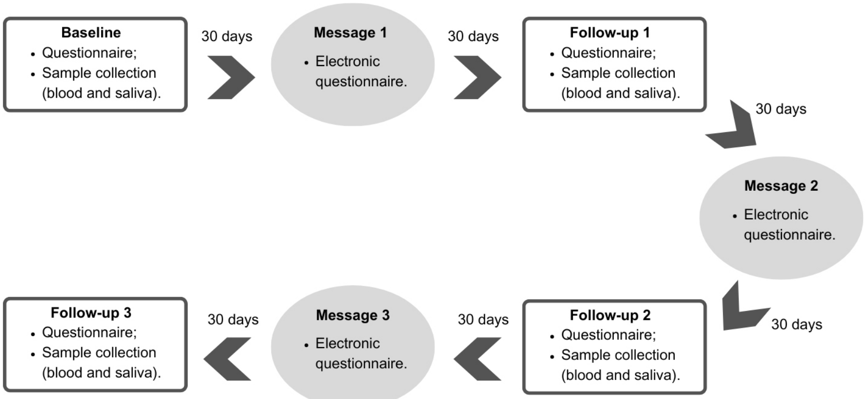

Supplementary Figure S1 - Blood donor procedures.

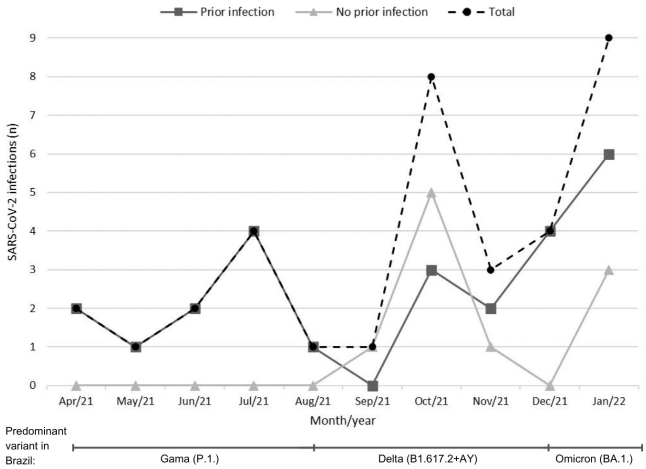

Supplementary Figure S2 - SARS-CoV-2 incident infections per month during the cohort follow-up. The predominant variants in each month were defined based on data from the Fiocruz Genomics Network[21].

This is an open-access article distributed under the terms of the Creative Commons Attribution License.
